# Supplementary material for: The Impact of Variation in the Toll-like Receptor 3 Gene on Epizootic Hemorrhagic Disease in Illinois Wild White-Tailed Deer (Odocoileus virginianus)
Source: Genes (Basel). 2023 Feb 8;14(2):426. doi: 10.3390/genes14020426 (PMC9956177; doi:10.3390/genes14020426)
Supplement: Supplementary file 1 [file genes-14-00426-s001.zip › genes-2116705 - Table S2.pdf]

Table S2. Polymorphic sites within the coding region of *TLR3* and haplotype frequencies for 84 white-tailed deer samples collected in Illinois. The number of chromosomes carrying each haplotype is listed for deer that were positive for epizootic hemorrhagic disease (EHD +) and for control deer (EHD -), with the overall frequency shown for each haplotype.

Haplotype	Nucleotide position within the *TLR3* coding region																																																																												Number of Chromosomes			
	38	39	108	111	121	175	225	347	363	366	391	661	672	705	718	851	856	873	903	908	966	995	1007	1030	1041	1044	1102	1117	1131	1143	1183	1184	1215	1228	1236	1260	1279	1317	1371	1386	1403	1434	1446	1458	1503	1576	1653	1690	1698	1759	1761	1806	1832	1867	1908	1993	2005	2018	2049	2085	2229	2265	2278	2284	2286	2353	2433	2476	2502	2529	2545	2578	2628	2634	2640	2668	2708	EHD (+)	EHD (-)	Total	Freq
Haplotype 01	C	G	C	T	C	C	C	C	C	C	C	A	G	C	G	G	T	C	C	A	T	G	G	C	T	C	T	A	A	G	C	G	C	C	G	C	G	C	C	C	A	C	C	G	G	T	C	C	C	C	T	T	T	A	G	A	A	G	A	G	C	A	A	C	C	A	A	C	C	C	T	C	T	C	G	T	T	4	8	12	0.0714
Haplotype 02	.	.	T	.	.	.	.	.	.	.	.	.	.	.	.	.	.	.	.	.	.	.	.	.	.	.	.	.	.	.	.	.	.	.	.	.	.	.	.	.	.	.	.	.	.	.	.	.	.	.	.	.	.	.	.	.	.	.	.	.	.	.	.	.	.	.	.	.	.	.	.	.	.	.	.	.	.	2	8	10	0.0595
Haplotype 03	.	.	.	.	.	.	.	.	.	.	.	.	.	.	.	.	**G**	.	.	.	.	.	.	.	.	.	.	.	.	.	.	.	.	.	A	.	**A**	.	.	.	.	.	.	.	.	**A**	.	.	T	.	.	.	.	.	.	.	.	.	.	.	.	.	.	.	.	.	.	.	.	.	C	T	.	.	.	.	.	2	5	7	0.0417
Haplotype 04	.	.	.	.	.	.	.	.	.	.	.	.	A	.	.	.	**G**	.	.	.	.	.	.	.	.	.	.	.	.	.	.	.	.	.	A	.	.	.	**A**	.	.	.	.	.	.	.	.	.	.	.	.	.	.	.	.	.	.	.	.	.	.	.	.	.	.	.	.	.	.	.	.	.	.	.	.	.	.	1	5	6	0.0357
Haplotype 05	.	.	.	.	.	.	T	.	.	.	.	.	.	.	.	.	**G**	.	.	.	.	.	.	.	.	.	.	.	G	.	.	.	.	.	.	T	.	.	.	T	.	.	.	.	.	.	.	.	.	.	.	.	.	.	.	.	.	.	G	.	.	.	.	.	.	.	.	T	.	.	.	.	.	.	.	.	.	4	2	6	0.0357
Haplotype 06	.	.	.	C	T	**T**	.	**T**	T	T	T	.	.	.	.	.	**G**	.	.	.	.	.	.	.	.	.	.	.	G	.	.	.	.	.	.	.	.	.	.	.	.	.	.	.	.	.	.	.	.	T	.	.	.	.	.	.	**G**	.	G	.	.	.	.	.	.	.	.	.	.	.	.	.	.	.	A	.	.	1	4	5	0.0298
Haplotype 07	.	.	.	.	.	.	.	.	.	.	.	.	.	.	.	.	**G**	.	.	.	.	.	.	.	.	.	.	.	.	.	.	.	.	.	A	.	**A**	.	.	.	.	.	.	.	.	**A**	.	.	T	.	.	.	.	**G**	.	.	.	.	.	.	.	.	.	.	.	.	.	.	.	.	C	T	.	.	.	.	.	2	2	4	0.0238
Haplotype 08	T	.	.	.	.	.	.	.	.	.	.	.	.	.	.	.	**G**	.	.	.	.	.	.	.	.	.	.	.	G	.	.	.	.	.	.	T	.	.	.	T	.	.	.	.	.	.	.	.	.	.	.	.	.	.	.	.	.	.	G	.	.	.	.	.	.	.	.	T	.	.	.	.	.	.	.	.	.	2	2	4	0.0238
Haplotype 09	.	**T**	.	.	.	.	.	.	.	.	.	.	.	.	.	.	**G**	.	.	.	.	.	.	.	.	.	.	.	.	.	.	.	.	.	.	.	.	.	.	.	.	.	.	C	.	.	.	.	.	.	.	.	.	.	.	.	.	.	.	.	.	.	.	.	.	.	.	.	.	.	.	.	.	.	.	.	.	3	1	4	0.0238
Haplotype 10	.	.	.	.	.	.	.	.	.	.	.	.	.	.	.	.	**G**	.	.	**T**	.	.	**C**	.	.	.	**C**	.	.	.	.	.	.	.	.	T	.	.	.	T	.	.	.	.	.	.	.	.	.	.	.	.	.	.	.	.	.	.	G	.	.	.	.	.	.	.	.	.	.	.	.	T	.	.	.	.	.	0	4	4	0.0238
Haplotype 11	.	**T**	.	.	.	.	.	.	.	.	.	.	.	.	.	.	**G**	.	.	.	.	.	.	.	.	.	.	.	.	.	**T**	.	.	.	.	.	.	.	.	.	.	.	.	C	.	.	.	.	.	.	.	.	.	.	.	.	.	.	.	.	.	.	.	.	.	.	.	.	.	.	.	.	.	.	.	.	.	0	4	4	0.0238
Haplotype 12	T	.	.	C	T	**T**	.	**T**	T	T	T	.	.	.	.	.	**G**	.	.	.	.	.	.	.	.	.	.	.	.	.	.	.	.	.	A	.	**A**	.	.	.	.	.	.	.	.	**A**	.	.	T	.	.	.	.	.	.	.	.	.	.	.	.	.	.	.	.	.	.	.	.	.	C	T	.	.	.	.	.	0	4	4	0.0238
Haplotype 13	T	.	.	.	.	.	.	.	.	.	.	.	.	.	.	.	.	.	.	.	.	.	.	.	.	.	.	.	.	.	.	**A**	T	.	.	.	.	.	.	.	.	.	.	.	.	.	.	.	.	.	.	.	.	.	.	.	.	.	.	.	.	.	.	.	.	.	.	.	.	.	.	.	.	.	.	.	.	2	1	3	0.0179
Haplotype 14	.	.	.	.	T	.	.	.	.	.	.	.	.	.	.	.	**G**	.	.	.	.	.	.	.	.	.	.	.	.	.	.	.	.	.	A	.	**A**	.	.	.	.	.	.	.	.	**A**	.	.	T	.	.	.	.	.	.	.	.	.	.	.	.	.	.	.	.	.	.	.	.	.	C	T	.	.	.	.	.	2	1	3	0.0179
Haplotype 15	T	.	.	.	.	.	.	.	.	.	.	.	.	.	.	.	.	.	.	.	.	.	.	.	.	.	.	.	G	.	.	.	.	.	.	.	.	.	.	.	.	.	.	.	.	.	.	.	.	.	**C**	.	.	.	.	.	**G**	**C**	G	.	.	.	.	**A**	.	.	G	T	.	.	.	.	.	.	.	.	.	2	1	3	0.0179
Haplotype 16	.	**T**	.	.	.	.	.	.	.	.	.	.	.	.	.	.	**G**	.	.	.	.	.	.	.	.	.	.	.	.	.	.	.	.	.	A	.	**A**	.	.	.	.	.	.	.	.	**A**	.	.	T	.	.	.	.	.	.	.	.	.	.	.	.	.	.	.	.	.	.	.	.	.	C	T	.	.	.	.	.	1	2	3	0.0179
Haplotype 17	.	.	.	.	.	.	.	.	.	.	.	.	A	.	.	.	**G**	.	.	.	.	.	.	.	.	.	.	.	.	.	.	.	.	.	A	.	.	.	**A**	.	.	.	.	.	.	.	.	.	.	T	.	.	.	.	.	.	**G**	.	G	.	.	.	.	.	.	.	.	T	.	.	.	T	.	.	.	.	.	1	2	3	0.0179
Haplotype 18	.	.	.	.	.	.	.	.	.	.	.	.	.	.	.	.	.	.	.	.	.	.	.	.	.	.	.	.	.	**C**	.	.	.	.	.	.	.	.	.	.	.	.	.	.	.	.	.	.	.	.	.	.	**A**	.	.	.	**G**	.	.	.	T	.	.	.	.	.	.	.	.	.	.	.	.	.	A	.	.	0	3	3	0.0179
Haplotype 19	.	.	.	.	.	.	.	.	.	.	.	.	.	.	.	**A**	.	.	.	.	.	.	**C**	.	.	.	**C**	.	.	.	.	.	.	.	.	T	.	.	.	T	.	.	.	.	.	.	.	.	.	.	.	.	.	.	.	.	.	.	G	.	.	.	.	.	.	.	.	.	.	.	.	T	.	.	.	.	.	0	2	2	0.0119
Haplotype 20	T	.	.	.	.	.	T	.	.	.	.	.	.	.	.	.	**G**	.	.	.	.	.	.	.	.	.	.	.	G	.	.	.	.	.	.	T	.	.	.	T	.	.	.	.	.	.	.	.	.	.	.	.	.	.	.	.	.	.	G	.	.	.	.	.	.	.	.	T	.	.	C	T	.	.	.	.	.	1	1	2	0.0119
Haplotype 21	.	.	.	.	.	.	T	.	.	.	.	.	.	.	.	.	**G**	.	.	.	.	.	.	.	.	.	.	.	G	.	.	.	.	.	.	T	.	.	.	T	.	.	.	.	.	.	.	.	.	.	.	.	.	.	.	.	.	.	G	.	.	.	.	.	.	.	.	T	.	.	C	T	.	.	.	.	.	0	2	2	0.0119
Haplotype 22	.	.	.	.	.	.	.	.	.	.	.	.	.	.	.	.	.	.	.	.	.	.	.	.	.	.	.	.	.	.	.	**A**	.	.	.	.	.	.	.	.	.	.	.	.	.	.	.	.	.	.	.	.	.	.	.	.	.	.	.	.	.	.	.	.	.	.	.	.	.	.	.	.	.	.	.	.	.	1	1	2	0.0119
Haplotype 23	.	.	T	.	.	.	.	.	.	.	.	.	A	.	.	.	**G**	.	.	.	.	.	.	**T**	.	T	.	.	G	.	.	.	.	.	.	.	.	.	.	.	.	.	.	.	.	.	.	.	.	T	.	.	.	.	.	.	**G**	.	G	.	.	.	.	.	.	.	.	.	.	.	.	.	.	.	.	.	.	1	1	2	0.0119
Haplotype 24	T	.	.	.	.	.	.	.	.	.	.	.	.	.	.	.	**G**	.	T	.	.	.	.	.	.	.	.	.	.	.	.	.	.	.	.	.	.	.	.	.	.	.	.	C	.	.	.	.	.	.	.	.	.	.	**C**	.	**G**	.	G	.	.	.	.	.	.	C	.	T	.	.	.	.	.	.	.	.	.	1	1	2	0.0119
Haplotype 25	.	**T**	.	.	.	.	.	.	.	.	.	.	.	.	.	.	**G**	.	.	.	.	.	.	.	.	.	.	.	.	.	.	.	.	.	.	.	.	.	.	.	.	.	.	C	.	.	T	.	.	.	.	.	.	.	.	.	.	.	.	.	.	.	.	.	.	.	.	.	.	.	.	.	.	.	.	.	.	1	1	2	0.0119
Haplotype 26	.	.	.	.	.	.	.	.	.	.	.	.	.	.	**A**	.	.	.	.	.	.	.	.	.	.	.	.	.	.	.	.	.	.	.	.	.	.	.	.	.	.	.	.	.	.	.	.	.	.	.	.	.	**A**	.	.	.	**G**	.	G	.	T	.	.	.	.	.	.	T	T	.	C	T	.	.	.	.	.	2	0	2	0.0119
Haplotype 27	T	.	.	.	.	.	.	.	.	.	.	.	.	.	.	.	**G**	.	.	.	.	.	.	.	.	.	.	.	G	.	.	.	.	.	.	T	.	.	.	T	.	.	.	.	.	.	.	.	.	.	.	.	.	.	.	.	.	.	G	.	.	.	.	.	.	.	.	T	.	.	.	T	.	.	.	.	.	0	2	2	0.0119
Haplotype 28	T	.	.	.	.	.	.	.	.	.	.	**G**	A	.	.	.	**G**	.	.	**T**	.	.	.	**T**	.	T	.	.	.	**C**	.	.	.	.	.	.	.	.	.	.	.	.	T	.	.	.	.	.	.	.	.	C	.	.	.	.	**G**	.	G	.	T	**C**	.	.	.	.	.	T	.	.	.	T	.	.	A	.	.	0	2	2	0.0119
Haplotype 29	.	.	.	.	.	.	T	.	.	.	.	.	.	.	.	.	.	.	.	.	.	.	.	.	.	.	.	.	G	.	.	.	.	.	.	.	.	.	.	.	.	.	.	.	.	.	.	.	.	T	.	.	.	.	.	.	**G**	.	G	.	T	.	.	.	.	.	.	T	.	.	C	T	.	.	.	.	.	0	2	2	0.0119
Haplotype 30	.	.	.	.	.	.	.	.	.	.	.	.	.	.	.	.	.	.	.	.	.	.	.	.	.	.	.	**G**	.	.	.	.	.	T	A	.	.	.	.	.	T	.	.	.	.	.	.	.	.	.	.	.	.	.	**C**	**C**	**G**	.	.	.	.	.	**G**	.	.	.	.	T	.	.	.	.	.	.	.	.	.	0	2	2	0.0119
Haplotype 31	T	.	.	.	.	.	.	.	.	.	.	.	.	.	.	.	.	.	.	.	.	.	.	.	.	.	.	.	G	.	.	.	.	.	.	T	.	.	.	.	.	.	.	.	.	.	.	.	.	.	.	C	.	.	.	.	**G**	.	G	.	T	**C**	.	.	.	.	.	T	.	.	.	T	.	.	.	.	.	0	2	2	0.0119
Haplotype 32	.	.	.	.	.	.	.	.	.	.	.	.	.	.	.	**A**	.	.	.	.	.	.	**C**	.	.	.	**C**	.	.	.	.	.	.	.	.	T	.	.	.	T	.	.	.	.	.	.	.	.	.	.	.	.	.	.	.	.	.	.	G	A	.	.	.	.	.	.	.	T	.	.	.	T	.	.	.	.	.	0	1	1	0.0060
Haplotype 33	.	.	.	.	.	.	.	.	.	.	.	.	.	.	.	**A**	.	.	.	.	.	.	**C**	.	.	.	**C**	.	.	.	.	.	.	.	.	T	.	.	.	T	.	.	.	.	.	.	.	.	.	.	.	.	.	.	.	.	.	.	G	.	.	.	.	.	.	.	.	T	.	.	C	T	.	.	.	.	.	1	0	1	0.0060
Haplotype 34	.	.	.	.	.	.	T	.	.	.	.	.	.	.	.	.	**G**	.	.	.	.	.	.	.	.	.	.	.	.	.	.	.	.	.	.	.	.	.	.	.	.	.	.	.	.	.	.	.	.	.	.	.	.	.	.	.	**G**	.	.	.	T	.	.	.	**T**	.	.	.	.	.	C	T	.	.	.	.	.	1	0	1	0.0060
Haplotype 35	.	.	.	.	.	.	.	.	.	.	.	.	.	.	.	**A**	.	.	.	.	.	.	**C**	.	.	.	**C**	.	.	.	.	.	.	.	.	T	.	.	.	T	.	.	.	.	.	.	.	.	.	.	.	.	.	.	.	.	.	.	G	A	.	.	.	.	.	.	.	T	.	.	C	T	.	.	.	.	.	1	0	1	0.0060
Haplotype 36	.	.	.	.	.	.	T	.	.	.	.	.	.	.	.	.	**G**	.	.	.	.	.	.	.	.	.	.	.	.	.	.	.	.	.	.	.	.	.	.	.	.	.	.	.	.	.	.	.	.	.	.	.	.	.	.	.	**G**	.	.	.	T	.	.	.	.	.	.	.	.	.	C	T	.	.	.	.	.	1	0	1	0.0060
Haplotype 37	T	.	.	.	.	.	.	.	.	.	.	.	.	.	.	.	**G**	.	.	.	.	.	.	.	.	.	.	.	G	.	.	.	.	.	.	T	.	.	.	T	.	.	.	.	.	.	.	.	.	.	.	.	.	.	.	.	.	.	G	.	.	.	.	.	.	.	.	T	.	.	C	T	.	.	.	.	.	1	0	1	0.0060
Haplotype 38	.	.	.	.	.	.	.	.	.	.	.	.	.	.	.	.	**G**	.	.	**T**	.	**A**	.	.	C	.	.	.	.	**C**	.	.	.	.	A	.	.	T	.	.	.	.	.	.	.	.	.	.	.	T	.	.	.	.	.	.	**G**	.	G	.	.	.	.	.	.	.	.	T	.	.	.	T	.	.	.	.	.	1	0	1	0.0060
Haplotype 39	.	.	.	.	.	.	T	.	.	.	.	.	.	.	.	.	**G**	.	.	.	.	.	.	.	.	.	.	.	.	.	.	.	.	.	.	.	.	.	.	.	.	.	.	.	.	.	.	.	.	.	.	.	.	.	.	.	**G**	.	.	.	T	.	.	.	**T**	.	.	.	.	.	.	.	.	.	.	.	.	1	0	1	0.0060
Haplotype 40	.	.	.	.	.	.	.	.	.	.	T	.	.	.	.	.	.	.	.	.	.	.	.	.	.	.	.	.	.	.	.	.	.	T	A	.	.	.	.	.	.	.	.	.	.	.	.	.	.	.	.	.	.	.	**C**	**C**	**G**	.	.	.	.	.	.	.	.	.	.	T	.	.	.	.	.	.	.	.	.	1	0	1	0.0060
Haplotype 41	.	.	.	.	.	.	T	.	.	.	.	.	.	.	.	.	.	.	.	.	.	.	.	.	.	.	.	.	G	.	.	.	.	.	.	.	.	.	.	.	.	.	.	.	.	.	.	.	.	T	.	.	.	.	.	.	**G**	.	G	.	T	.	.	.	.	.	.	T	.	.	.	.	.	.	.	.	.	1	0	1	0.0060
Haplotype 42	.	.	.	.	.	.	.	.	.	.	.	.	.	.	.	.	**G**	.	.	.	.	.	.	.	.	.	.	.	G	.	.	.	.	.	.	.	.	.	.	.	.	.	.	.	.	.	.	.	.	T	.	.	.	.	.	.	**G**	.	G	.	.	.	.	.	.	.	.	.	.	.	.	.	.	.	A	.	.	0	1	1	0.0060
Haplotype 43	.	.	.	C	T	**T**	.	.	.	.	.	.	.	.	.	.	**G**	.	.	.	.	.	.	.	.	.	.	.	.	.	.	.	.	.	A	.	**A**	.	.	.	.	.	.	.	.	**A**	.	.	T	.	.	.	.	.	.	.	.	.	.	.	.	.	.	.	.	.	.	.	.	.	C	T	.	.	.	.	.	0	1	1	0.0060
Haplotype 44	.	.	.	.	.	.	.	.	.	.	.	.	.	.	.	**A**	.	A	.	.	.	.	.	**T**	.	.	.	.	.	.	.	.	.	.	.	.	.	.	.	.	.	.	.	.	.	.	.	.	.	.	.	.	.	.	.	.	.	.	.	.	.	.	.	.	.	.	.	.	.	.	.	.	.	.	.	.	.	0	1	1	0.0060
Haplotype 45	.	.	.	.	.	.	T	.	.	.	.	.	.	T	.	.	.	.	.	.	.	.	.	.	.	.	.	.	.	.	.	.	.	.	.	.	**A**	.	.	.	.	.	.	.	.	**A**	.	.	T	.	.	.	.	.	.	.	.	.	.	.	.	.	.	.	.	.	.	.	.	.	C	T	.	.	.	.	.	1	0	1	0.0060
Haplotype 46	.	.	.	.	.	.	.	.	.	.	.	.	.	.	.	.	**G**	.	.	**T**	.	**A**	.	.	C	.	.	.	.	**C**	.	.	.	.	.	.	.	.	.	.	.	.	.	.	.	.	.	.	.	.	.	.	.	.	.	.	.	.	.	.	.	.	.	.	.	.	.	.	.	.	.	.	.	.	.	.	.	1	0	1	0.0060
Haplotype 47	.	.	.	.	.	.	.	.	.	.	.	.	.	.	.	.	**G**	.	.	**T**	.	**A**	.	.	C	.	.	.	.	**C**	.	.	.	.	A	.	.	T	.	.	.	.	.	.	.	.	.	**T**	.	.	.	.	.	.	.	.	**G**	.	G	.	.	.	.	.	.	.	.	T	.	.	.	T	.	.	.	.	.	1	0	1	0.0060
Haplotype 48	.	.	.	.	.	.	.	.	.	.	.	.	.	.	.	.	**G**	.	.	.	C	.	.	**T**	.	T	.	.	G	.	.	.	.	.	A	.	.	.	.	.	.	.	.	.	.	.	.	.	.	T	.	.	.	.	.	.	**G**	.	G	.	.	.	.	.	.	.	.	T	.	.	.	T	.	.	.	.	.	1	0	1	0.0060
Haplotype 49	.	.	.	C	.	.	.	.	.	.	.	.	.	.	.	**A**	.	A	.	.	.	.	.	**T**	.	.	.	.	.	.	.	.	.	.	.	.	.	.	.	.	.	.	.	.	.	.	.	.	.	.	.	.	.	.	.	.	.	.	.	.	.	.	.	.	.	.	.	.	.	.	C	T	.	.	.	.	.	1	0	1	0.0060
Haplotype 50	.	.	.	C	.	.	.	.	.	.	.	.	.	T	.	.	.	.	.	.	.	.	.	.	.	.	.	.	.	.	.	.	.	.	A	.	.	.	.	.	.	.	.	.	.	.	.	.	.	.	.	.	**A**	.	.	.	**G**	.	G	.	T	.	.	.	.	.	.	T	.	.	C	T	.	.	.	.	.	1	0	1	0.0060
Haplotype 51	.	.	.	.	.	.	T	.	.	.	.	.	.	.	.	.	**G**	.	.	.	.	.	.	.	.	.	.	.	G	.	.	.	.	.	.	T	.	.	.	T	.	.	.	.	.	.	.	.	.	.	.	.	.	.	.	.	.	.	G	.	.	.	.	.	.	.	.	.	.	.	.	.	.	.	.	.	.	1	0	1	0.0060
Haplotype 52	.	.	.	.	.	.	.	**T**	T	T	.	.	A	.	.	.	**G**	.	.	.	.	.	.	.	.	.	.	.	.	.	.	.	.	.	A	.	.	.	**A**	.	.	.	.	.	.	.	.	.	.	T	.	.	.	.	.	.	**G**	.	G	.	.	.	.	.	.	.	.	T	.	.	.	T	.	.	.	.	.	0	1	1	0.0060
Haplotype 53	.	**T**	.	.	.	.	.	.	.	.	.	.	.	.	.	.	**G**	.	.	**T**	.	**A**	.	.	C	.	.	.	.	**C**	.	.	.	.	A	.	.	T	.	.	.	.	.	.	A	.	.	.	.	T	.	.	.	.	.	.	**G**	.	G	.	.	.	.	.	.	.	.	T	.	.	.	T	.	.	.	.	.	0	1	1	0.0060
Haplotype 54	T	.	.	.	.	.	.	.	.	.	.	.	.	.	.	.	.	A	.	.	.	.	.	.	.	.	.	.	.	.	.	.	.	.	A	.	.	.	.	.	.	.	.	.	.	.	.	.	.	.	.	C	.	.	.	.	**G**	.	G	.	T	.	.	.	.	.	.	T	.	.	.	T	.	.	.	.	.	0	1	1	0.0060
Haplotype 55	T	.	.	.	T	**T**	.	**T**	.	.	.	**G**	A	.	.	.	**G**	.	.	**T**	.	.	.	**T**	.	T	.	.	.	**C**	.	.	.	.	.	.	.	.	.	.	.	.	T	.	.	.	.	.	.	.	.	C	.	.	.	.	**G**	.	G	.	T	.	.	.	.	.	.	T	.	.	.	T	.	.	.	.	.	0	1	1	0.0060
Haplotype 56	.	.	.	.	.	.	.	.	.	.	.	.	.	.	.	.	.	.	.	.	.	.	.	.	.	.	.	.	.	.	.	.	.	.	.	.	.	.	.	.	.	.	.	.	.	**A**	.	.	.	.	.	.	.	.	.	.	.	.	.	.	.	.	.	.	.	.	.	.	.	.	.	.	.	.	.	.	.	0	1	1	0.0060
Haplotype 57	T	.	.	.	.	.	.	.	.	.	.	.	.	.	.	**A**	.	.	.	.	.	.	**C**	.	.	.	.	.	.	.	.	.	.	.	A	.	.	.	.	T	.	.	.	.	.	.	.	.	.	.	.	.	.	.	**C**	.	**G**	.	G	.	.	.	.	.	.	.	.	T	.	.	.	.	.	.	.	.	.	0	1	1	0.0060
Haplotype 58	T	.	.	.	T	.	.	.	.	.	.	.	.	.	.	.	.	.	.	.	.	.	.	.	.	.	.	.	.	.	.	.	.	.	.	.	.	.	.	.	.	.	.	.	.	.	.	.	.	.	.	.	.	.	.	.	.	.	.	.	.	.	.	.	.	.	.	.	.	.	.	.	.	.	.	.	.	0	1	1	0.0060
Haplotype 59	.	.	.	.	.	.	.	.	.	.	T	.	.	.	.	.	.	.	.	.	.	.	.	.	.	.	.	.	G	.	.	.	.	.	.	.	.	.	.	.	.	.	.	.	.	.	.	.	.	.	**C**	.	.	.	.	.	**G**	**C**	G	.	.	.	.	**A**	.	.	G	T	.	.	C	.	.	.	.	.	.	0	1	1	0.0060
Haplotype 60	.	.	.	.	.	.	.	.	.	.	.	.	.	.	.	.	**G**	.	T	.	.	.	.	.	.	.	.	.	.	.	.	.	.	.	A	.	.	.	.	.	G	.	.	.	.	.	.	.	.	.	.	.	.	.	.	.	**G**	.	G	.	T	.	.	.	.	.	.	T	.	.	.	.	.	.	.	.	.	0	1	1	0.0060
Haplotype 61	.	.	.	.	.	.	.	.	.	.	.	.	.	.	.	.	**G**	.	T	.	.	.	.	.	.	.	.	.	.	.	.	.	.	.	A	.	.	.	.	.	G	.	.	.	.	.	.	.	.	.	.	.	.	.	.	.	**G**	.	G	.	T	.	.	.	.	.	.	T	.	.	.	T	.	.	.	.	.	0	1	1	0.0060
Haplotype 62	.	.	.	.	.	.	.	.	.	.	.	.	.	.	.	**A**	.	.	.	.	.	.	**C**	.	.	.	**C**	.	.	.	.	.	.	.	.	T	.	.	.	T	.	.	.	.	.	.	.	.	.	.	.	.	.	.	.	.	**G**	.	.	.	T	.	.	.	**T**	.	.	.	.	.	.	.	.	.	.	.	.	0	1	1	0.0060
Haplotype 63	.	.	.	C	T	**T**	.	**T**	.	.	.	.	.	.	.	.	**G**	.	.	.	.	.	.	.	.	.	.	.	.	.	.	.	.	.	A	.	**A**	.	.	.	.	.	.	.	.	**A**	.	.	T	.	.	.	.	.	.	.	.	.	.	.	.	.	.	.	.	.	.	.	.	.	C	T	.	.	.	.	.	0	1	1	0.0060
Haplotype 64	.	.	.	.	.	.	.	.	.	.	.	.	.	.	.	**A**	.	.	.	.	.	.	**C**	.	.	.	.	.	.	.	.	.	.	.	A	.	.	.	.	T	.	.	.	.	.	.	.	.	.	.	.	.	.	.	**C**	.	**G**	.	G	.	.	.	.	.	.	.	.	T	.	.	.	.	.	.	.	.	.	0	1	1	0.0060
Haplotype 65	.	.	.	.	.	**T**	.	.	.	.	.	.	.	.	.	**A**	.	.	.	.	.	.	**C**	.	.	.	.	.	.	.	.	.	.	.	A	.	.	.	.	T	.	.	.	.	.	.	.	.	.	.	.	.	.	.	**C**	.	**G**	.	G	.	.	.	.	.	.	.	.	T	.	.	.	.	.	.	.	.	.	0	1	1	0.0060
Haplotype 66	.	.	.	.	.	.	.	.	.	.	.	.	A	.	.	.	**G**	.	.	.	.	.	.	.	.	.	.	.	.	.	.	.	.	.	A	.	.	.	**A**	.	.	.	.	.	.	.	.	.	.	.	.	.	.	.	.	.	**G**	.	.	.	.	.	.	.	.	.	.	.	.	.	.	.	.	.	.	.	.	0	1	1	0.0060
Haplotype 67	T	.	.	.	.	.	T	.	.	.	.	.	A	.	.	**A**	.	.	.	.	.	.	**C**	.	.	.	**C**	.	.	.	.	.	.	.	.	T	.	.	.	T	.	.	.	.	.	.	.	.	.	.	.	.	.	.	.	.	.	.	G	.	.	.	.	.	.	.	.	T	.	.	.	.	.	.	A	.	.	0	1	1	0.0060
Haplotype 68	.	.	.	.	.	.	.	.	.	.	.	.	.	.	.	**A**	.	A	.	.	.	.	.	**T**	.	.	.	.	.	.	.	.	.	.	.	.	.	.	.	.	.	.	.	.	.	.	.	.	.	.	.	.	.	.	.	.	.	.	.	.	.	.	.	.	.	.	.	.	.	.	C	T	.	.	.	.	.	0	1	1	0.0060
Haplotype 69	T	.	.	C	T	**T**	.	**T**	T	T	T	.	.	.	.	.	.	.	.	.	.	.	.	.	.	.	.	.	G	.	.	.	.	.	.	.	.	.	.	.	.	.	.	.	.	.	.	.	.	.	**C**	.	.	.	.	.	**G**	**C**	G	.	.	.	.	**A**	.	.	G	T	.	.	.	.	.	.	.	.	.	0	1	1	0.0060
Haplotype 70	.	.	.	.	.	.	.	.	.	.	.	.	.	.	.	**A**	.	.	.	.	.	.	**C**	.	.	.	**C**	.	.	.	.	.	.	.	.	T	.	.	.	T	.	.	.	.	.	.	.	.	.	.	.	.	.	.	.	.	.	.	G	.	.	.	.	.	.	.	.	T	.	.	.	.	.	.	.	.	.	0	1	1	0.0060
Haplotype 71	.	.	.	.	.	**T**	T	**T**	.	.	.	.	.	T	.	.	.	.	.	.	.	.	.	.	.	.	.	.	.	.	.	.	.	.	A	.	**A**	.	.	.	.	.	.	.	.	**A**	.	.	T	.	.	.	.	.	.	.	.	.	.	.	.	.	.	.	.	.	.	.	.	.	C	T	.	.	.	.	.	0	1	1	0.0060
Haplotype 72	.	.	.	.	.	.	.	.	.	.	.	.	.	.	.	.	**G**	.	.	.	.	.	.	.	.	.	.	.	G	.	.	.	.	.	.	T	.	.	.	T	.	.	.	.	.	.	.	.	.	.	.	.	.	.	.	.	.	.	G	.	.	.	.	.	.	.	.	T	.	.	C	T	.	.	.	.	.	0	1	1	0.0060
Haplotype 73	.	.	.	.	.	.	.	.	.	.	.	.	.	.	.	**A**	.	.	.	.	.	.	**C**	.	.	.	**C**	.	.	.	.	.	.	.	.	.	.	.	.	T	.	.	.	.	.	.	.	.	.	.	.	.	.	.	.	.	.	.	G	.	.	.	.	.	.	.	.	T	.	.	.	.	.	.	.	.	.	0	1	1	0.0060
Haplotype 74	.	.	.	.	.	.	.	.	.	.	.	.	.	.	.	.	.	.	.	.	.	.	.	.	.	.	.	.	.	.	.	.	.	.	.	.	.	.	.	.	.	.	.	.	.	.	.	.	.	T	.	.	.	.	.	.	**G**	.	G	.	.	.	.	.	.	.	.	T	.	.	.	T	.	.	.	.	.	0	1	1	0.0060
Haplotype 75	.	.	.	.	.	.	.	.	.	.	.	.	.	.	.	.	**G**	.	T	.	.	.	.	.	.	.	.	.	.	.	.	.	.	.	.	.	.	.	.	.	.	.	.	C	.	.	.	.	.	.	.	.	.	.	**C**	.	**G**	.	G	.	.	.	.	.	.	C	.	T	.	.	.	.	.	.	.	.	.	0	1	1	0.0060
Haplotype 76	.	.	.	.	.	.	.	.	.	.	.	.	.	.	.	.	**G**	.	.	**T**	.	.	**C**	.	.	.	.	.	.	.	.	.	.	.	A	.	**A**	.	.	.	.	.	.	.	.	**A**	.	.	T	.	.	.	.	.	.	.	.	.	.	.	.	.	.	.	.	.	.	.	T	T	.	.	C	T	.	**C**	**C**	0	1	1	0.0060
Haplotype 77	T	.	.	.	T	**T**	.	**T**	T	T	.	.	.	.	.	.	.	.	T	.	.	.	.	.	.	.	.	.	G	.	.	.	.	.	.	T	.	.	.	T	.	.	.	.	.	.	.	.	.	.	.	.	.	.	.	.	.	.	G	.	.	.	.	.	.	.	.	T	T	T	.	.	C	T	.	**C**	**C**	0	1	1	0.0060
Haplotype 78	.	.	.	.	.	.	.	.	.	.	.	.	.	.	.	.	.	.	.	.	.	.	.	.	.	.	.	.	.	.	.	.	.	.	.	.	.	.	.	.	.	.	.	.	.	**A**	.	.	.	.	.	.	.	.	.	.	.	.	.	.	.	.	.	.	.	.	.	.	.	.	.	T	.	.	.	.	.	0	1	1	0.0060
Haplotype 79	T	.	.	.	.	.	.	.	.	.	.	**G**	A	.	.	.	.	.	.	.	.	.	.	.	.	.	.	.	.	.	.	.	.	.	A	.	.	.	.	.	.	.	.	.	.	.	.	.	.	T	.	.	.	.	.	.	.	.	.	.	.	.	.	.	.	.	.	.	.	.	.	T	.	.	.	.	.	0	1	1	0.0060
Haplotype 80	T	.	.	.	T	.	.	**T**	T	T	.	.	.	.	.	.	.	.	.	.	.	.	.	.	.	.	.	.	.	.	.	**A**	T	.	.	.	.	.	.	.	.	.	.	.	.	.	.	.	.	.	.	.	.	.	.	.	.	.	.	.	.	.	.	.	.	.	.	.	.	.	.	.	.	.	.	.	.	0	1	1	0.0060
Haplotype 81	.	.	.	.	.	.	.	.	.	.	.	.	.	.	.	.	.	.	.	.	.	.	.	.	.	.	.	.	G	.	.	.	.	.	.	.	.	.	.	.	.	.	.	.	.	.	.	.	.	.	**C**	.	.	.	.	.	**G**	**C**	G	.	.	.	.	**A**	.	.	G	T	.	.	.	.	.	.	.	.	.	0	1	1	0.0060
Haplotype 82	T	.	.	C	T	**T**	.	**T**	T	T	T	.	.	.	.	.	**G**	.	.	.	.	.	.	.	.	.	.	.	.	.	.	.	.	.	A	.	**A**	.	.	.	.	.	.	.	.	**A**	.	.	T	.	.	.	.	.	.	.	.	.	.	.	.	.	.	.	.	.	.	.	.	.	.	T	.	.	.	.	.	0	1	1	0.0060
Haplotype 83	.	.	.	.	.	.	.	**T**	.	.	.	.	.	.	.	**A**	.	.	.	.	.	.	**C**	.	.	.	.	.	.	.	.	.	.	.	A	.	.	.	.	T	.	.	.	.	.	.	.	.	.	.	.	.	.	.	.	.	**G**	.	G	.	.	.	.	.	.	.	.	T	.	.	.	T	.	.	.	.	.	0	1	1	0.0060
Haplotype 84	T	.	.	C	T	**T**	.	**T**	T	T	T	.	.	.	.	.	**G**	.	.	.	.	.	.	.	.	.	.	.	.	.	.	.	.	.	A	.	**A**	.	.	.	.	.	.	.	.	**A**	.	.	T	.	.	.	.	.	.	.	.	.	.	.	.	.	.	.	.	.	.	.	.	.	C	T	.	T	.	.	.	0	1	1	0.0060
Haplotype 85	.	.	.	.	.	.	T	.	.	.	.	.	.	.	.	.	.	.	.	.	.	.	.	.	.	.	.	.	G	.	.	.	.	.	.	.	.	.	.	.	.	.	.	.	.	.	.	.	.	.	**C**	.	.	.	.	.	**G**	**C**	G	.	.	.	.	**A**	.	.	G	T	.	.	.	T	.	.	.	.	.	0	1	1	0.0060
**Exon/Total**	Exon 2											Exon 4																																																												Exon 5								**52**	**116**	**168**	**1**

Numbers across the top refer to the nucleotide position within the coding region of *TLR3* . Nucleotide character states that are identical to those of Haplotype 01 are shown as dots, while for nucleotide positions that differ from Haplotype 01, the character state is shown. Nonsynonymous mutations are identified in boldface; all other substitutions are synonymous. The exons of each SNP are indicated at the bottom of the table.
