# Supplementary material for: The Impact of Variation in the Toll-like Receptor 3 Gene on Epizootic Hemorrhagic Disease in Illinois Wild White-Tailed Deer (Odocoileus virginianus)
Source: Genes (Basel). 2023 Feb 8;14(2):426. doi: 10.3390/genes14020426 (PMC9956177; doi:10.3390/genes14020426)
Supplement: Supplementary file 1 [file genes-14-00426-s001.zip › genes-2116705 - Table S3.pdf]

Table S3. Amino acid variation in the TLR3 protein among 84 white-tailed deer collected in Illinois. The number of chromosomes encoding each protein variant are listed for epizootic hemorrhagic disease positive (EHD +) and for control (EHD -) deer, along with the total frequency per variant.

Protein variant	Positions of non-synonymous nucleotide mutations, and of codons																																Number of chromosomes encoding protein variant			
Nucleotide:	39	175	347	661	718	851	856	908	995	1007	1030	1102	1117	1143	1183/1184	1279	1371	1403	1576	1690	1761	1832	1867	1908	1993	2005	2018	2265	2278	2284	2668	2708				
Codon:	13	59	116	221	240	284	286	303	332	336	344	368	373	381	395	427	457	468	526	564	587	611	623	636	665	669	673	755	760	762	890	903	EHD (+)	EHD (-)	Total	Freq.
TLR3 01*	S	L	S	I	E	R	S	Y	R	R	L	F	R	R	R	A	D	K	L	R	R	V	I	R	T	I	R	E	I	H	Y	V	6	16	22	0.1310
TLR3 03*	.	.	.	.	.	.	A	.	.	.	.	.	.	.	.	T	.	.	M	.	.	.	.	.	.	.	.	.	.	.	.	.	5	8	13	0.0774
TLR3 04	.	.	.	.	.	.	A	.	.	.	.	.	.	.	.	.	E	.	.	.	.	.	.	.	.	.	.	.	.	.	.	.	1	5	6	0.0357
TLR3 05*	.	.	.	.	.	.	A	.	.	.	.	.	.	.	.	.	.	.	.	.	.	.	.	.	.	.	.	.	.	.	.	.	9	7	16	0.0952
TLR3 06	.	**F**	**F**	.	.	.	A	.	.	.	.	.	.	.	.	.	.	.	.	.	C	.	.	.	.	V	.	.	.	.	.	.	1	4	5	0.0298
TLR3 07	.	.	.	.	.	.	A	.	.	.	.	.	.	.	.	T	.	.	M	.	.	.	V	.	.	.	.	.	.	.	.	.	2	2	4	0.0238
TLR3 08*	L	.	.	.	.	.	A	.	.	.	.	.	.	.	.	.	.	.	.	.	.	.	.	.	.	.	.	.	.	.	.	.	4	5	9	0.0536
TLR3 10	.	.	.	.	.	.	A	F	.	T	.	L	.	.	.	.	.	.	.	.	.	.	.	.	.	.	.	.	.	.	.	.	0	4	4	0.0238
TLR3 11	.	.	.	.	.	.	A	.	.	.	.	.	.	.	W	.	.	.	.	.	.	.	.	.	.	.	.	.	.	.	.	.	0	4	4	0.0238
TLR3 12*	L	**F**	**F**	.	.	.	A	.	.	.	.	.	.	.	.	T	.	.	M	.	.	.	.	.	.	.	.	.	.	.	.	.	0	6	6	0.0357
TLR3 13	L	.	.	.	.	.	.	.	.	.	.	.	.	.	Q	.	.	.	.	.	.	.	.	.	.	.	.	.	.	.	.	.	2	1	3	0.0179
TLR3 15	L	.	.	.	.	.	.	.	.	.	.	.	.	.	.	.	.	.	.	.	.	.	.	.	.	V	T	.	.	N	.	.	2	1	3	0.0179
TLR3 17	.	.	.	.	.	.	A	.	.	.	.	.	.	.	.	.	E	.	.	.	C	.	.	.	.	V	.	.	.	.	.	.	1	2	3	0.0179
TLR3 18	.	.	.	.	.	.	.	.	.	.	.	.	.	S	.	.	.	.	.	.	.	E	.	.	.	V	.	.	.	.	.	.	0	3	3	0.0179
TLR3 19*	.	.	.	.	.	H	.	.	.	T	.	L	.	.	.	.	.	.	.	.	.	.	.	.	.	.	.	.	.	.	.	.	2	5	7	0.0417
TLR3 22	.	.	.	.	.	.	.	.	.	.	.	.	.	.	Q	.	.	.	.	.	.	.	.	.	.	.	.	.	.	.	.	.	1	1	2	0.0119
TLR3 23*	.	.	.	.	.	.	A	.	.	.	F	.	.	.	.	.	.	.	.	.	C	.	.	.	.	V	.	.	.	.	.	.	2	1	3	0.0179
TLR3 24	L	.	.	.	.	.	A	.	.	.	.	.	.	.	.	.	.	.	.	.	.	.	.	S	.	V	.	.	.	.	.	.	1	1	2	0.0119
TLR3 26	.	.	.	.	K	.	.	.	.	.	.	.	.	.	.	.	.	.	.	.	.	E	.	.	.	V	.	.	.	.	.	.	2	0	2	0.0119
TLR3 28	L	.	.	V	.	.	A	F	.	.	F	.	.	S	.	.	.	.	.	.	.	.	.	.	.	V	.	D	.	.	.	.	0	2	2	0.0119
TLR3 29*	.	.	.	.	.	.	.	.	.	.	.	.	.	.	.	.	.	.	.	.	C	.	.	.	.	V	.	.	.	.	.	.	1	3	4	0.0238
TLR3 30	.	.	.	.	.	.	.	.	.	.	.	.	G	.	.	.	.	.	.	.	.	.	.	S	P	V	.	.	V	.	.	.	0	2	2	0.0119
TLR3 31	L	.	.	.	.	.	.	.	.	.	.	.	.	.	.	.	.	.	.	.	.	.	.	.	.	V	.	D	.	.	.	.	0	2	2	0.0119
TLR3 34*	.	.	.	.	.	.	A	.	.	.	.	.	.	.	.	.	.	.	.	.	.	.	.	.	.	V	.	.	.	.	.	.	3	0	3	0.0179
TLR3 38*	.	.	.	.	.	.	A	F	Q	.	.	.	.	S	.	.	.	.	.	.	C	.	.	.	.	V	.	.	.	.	.	.	1	1	2	0.0119
TLR3 40	.	.	.	.	.	.	.	.	.	.	.	.	.	.	.	.	.	.	.	.	.	.	.	S	P	V	.	.	.	.	.	.	1	0	1	0.0060
TLR3 42	.	.	.	.	.	.	A	.	.	.	.	.	.	.	.	.	.	.	.	.	C	.	.	.	.	V	.	.	.	.	.	.	0	1	1	0.0060
TLR3 43	.	**F**	.	.	.	.	A	.	.	.	.	.	.	.	.	T	.	.	M	.	.	.	.	.	.	.	.	.	.	.	.	.	0	1	1	0.0060
TLR3 44*	.	.	.	.	.	H	.	.	.	.	F	.	.	.	.	.	.	.	.	.	.	.	.	.	.	.	.	.	.	.	.	.	1	2	3	0.0179
TLR3 45	.	.	.	.	.	.	.	.	.	.	.	.	.	.	.	T	.	.	M	.	.	.	.	.	.	.	.	.	.	.	.	.	1	0	1	0.0060
TLR3 46	.	.	.	.	.	.	A	F	Q	.	.	.	.	S	.	.	.	.	.	.	.	.	.	.	.	.	.	.	.	.	.	.	1	0	1	0.0060
TLR3 47	.	.	.	.	.	.	A	F	Q	.	.	.	.	S	.	.	.	.	.	C	.	.	.	.	.	V	.	.	.	.	.	.	1	0	1	0.0060
TLR3 50	.	.	.	.	.	.	.	.	.	.	.	.	.	.	.	.	.	.	.	.	.	E	.	.	.	V	.	.	.	.	.	.	1	0	1	0.0060
TLR3 52	.	.	**F**	.	.	.	A	.	.	.	.	.	.	.	.	.	E	.	.	.	C	.	.	.	.	V	.	.	.	.	.	.	0	1	1	0.0060
TLR3 54	L	.	.	.	.	.	.	.	.	.	.	.	.	.	.	.	.	.	.	.	.	.	.	.	.	V	.	.	.	.	.	.	0	1	1	0.0060
TLR3 55	L	**F**	**F**	V	.	.	A	F	.	.	F	.	.	S	.	.	.	.	.	.	.	.	.	.	.	V	.	.	.	.	.	.	0	1	1	0.0060
TLR3 56*	.	.	.	.	.	.	.	.	.	.	.	.	.	.	.	.	.	.	M	.	.	.	.	.	.	.	.	.	.	.	.	.	0	2	2	0.0119
TLR3 57	L	.	.	.	.	H	.	.	.	T	.	.	.	.	.	.	.	.	.	.	.	.	.	S	.	V	.	.	.	.	.	.	0	1	1	0.0060
TLR3 58	L	.	.	.	.	.	.	.	.	.	.	.	.	.	.	.	.	.	.	.	.	.	.	.	.	.	.	.	.	.	.	.	0	1	1	0.0060
TLR3 59*	.	.	.	.	.	.	.	.	.	.	.	.	.	.	.	.	.	.	.	.	.	.	.	.	.	V	T	.	.	N	.	.	0	3	3	0.0179
TLR3 60*	.	.	.	.	.	.	A	.	.	.	.	.	.	.	.	.	.	R	.	.	.	.	.	.	.	V	.	.	.	.	.	.	0	2	2	0.0119
TLR3 62	.	.	.	.	.	H	.	.	.	T	.	L	.	.	.	.	.	.	.	.	.	.	.	.	.	V	.	.	.	.	.	.	0	1	1	0.0060
TLR3 63	.	**F**	**F**	.	.	.	A	.	.	.	.	.	.	.	.	T	.	.	M	.	.	.	.	.	.	.	.	.	.	.	.	.	0	1	1	0.0060
TLR3 64	.	.	.	.	.	H	.	.	.	T	.	.	.	.	.	.	.	.	.	.	.	.	.	S	.	V	.	.	.	.	.	.	0	1	1	0.0060
TLR3 65	.	**F**	.	.	.	H	.	.	.	T	.	.	.	.	.	.	.	.	.	.	.	.	.	S	.	V	.	.	.	.	.	.	0	1	1	0.0060
TLR3 66	.	.	.	.	.	.	A	.	.	.	.	.	.	.	.	.	E	.	.	.	.	.	.	.	.	V	.	.	.	.	.	.	0	1	1	0.0060
TLR3 67	L	.	.	.	.	H	.	.	.	T	.	L	.	.	.	.	.	.	.	.	.	.	.	.	.	.	.	.	.	.	.	.	0	1	1	0.0060
TLR3 69	L	**F**	**F**	.	.	.	.	.	.	.	.	.	.	.	.	.	.	.	.	.	.	.	.	.	.	V	T	.	.	N	.	.	0	1	1	0.0060
TLR3 71	.	**F**	**F**	.	.	.	.	.	.	.	.	.	.	.	.	T	.	.	M	.	.	.	.	.	.	.	.	.	.	.	.	.	0	1	1	0.0060
TLR3 75	.	.	.	.	.	.	A	.	.	.	.	.	.	.	.	.	.	.	.	.	.	.	.	S	.	V	.	.	.	.	.	.	0	1	1	0.0060
TLR3 76	.	.	.	.	.	.	A	F	.	T	.	.	.	.	.	T	.	.	M	.	.	.	.	.	.	.	.	.	.	.	H	A	0	1	1	0.0060
TLR3 77	L	**F**	**F**	.	.	.	.	.	.	.	.	.	.	.	.	.	.	.	.	.	.	.	.	.	.	.	.	.	.	.	H	A	0	1	1	0.0060
TLR3 79	L	.	.	V	.	.	.	.	.	.	.	.	.	.	.	.	.	.	.	.	C	.	.	.	.	.	.	.	.	.	.	.	0	1	1	0.0060
TLR3 80	L	.	**F**	.	.	.	.	.	.	.	.	.	.	.	Q	.	.	.	.	.	.	.	.	.	.	.	.	.	.	.	.	.	0	1	1	0.0060
TLR3 83	.	.	**F**	.	.	H	.	.	.	T	.	.	.	.	.	.	.	.	.	.	.	.	.	.	.	V	.	.	.	.	.	.	0	1	1	0.0060
Toal of reference AA	132	150	149	164	166	152	74	156	164	150	159	155	166	158	158	140	157	166	138	167	148	162	164	159	165	112	161	164	166	161	166	166				
Total of variant AA	36	18	19	4	2	16	94	12	4	18	9	13	2	10	F:6, W:4	28	11	2	30	1	20	6	4	9	3	56	7	4	2	7	2	2				
**Exon/Total**																																	**52**	**116**	**168**	**1**

The row labelled "nucleotide" indicates the position of the non-synonymous mutation in the coding region of *TLR3* , while the row labelled "codon" indicates the number of the codon. Translated amino acid (AA) residues that match those of protein variant TLR3 01 at the same position are shown as dots and also designated as a reference AA, while those that differ from protein variant TLR3 01 are shown as the substituted amino acid and included in the total of variant AA. The two amino acid substitutions associated with a lower incidence of EHD are in boldface. Protein variants
