# Supplementary material for: The Impact of Variation in the Toll-like Receptor 3 Gene on Epizootic Hemorrhagic Disease in Illinois Wild White-Tailed Deer (Odocoileus virginianus)
Source: Genes (Basel). 2023 Feb 8;14(2):426. doi: 10.3390/genes14020426 (PMC9956177; doi:10.3390/genes14020426)
Supplement: Supplementary file 1 [file genes-14-00426-s001.zip › genes-2116705-supplementary Figure S1 and Table S1.pdf]

Supplementary Figure

Figure S1: Aligned protein translation of the mRNA sequences of *TLR3* of human (NM_003265) and cattle (NM_001008664), and the protein translation of the most common haplotype (Haplotype 1) found by our study. The amino acid sequence that we detected in white-tailed deer is homologous to those of two well-studied species. This alignment was made using Clustal Omega (https://www.ebi.ac.uk/Tools/msa/clustalo/).

```
TLR3_Human              MRQTL-PCIYFWGGLLPFGMLCASSTTKCTVSHEVADCSHLKLTQVPDDLPTNITVLNLT  59
TLR3_Cattle             MSRPLPYHIHFFSGLLTCWILCTSSAHKCTVRHEVADCSHLKLTQIPDDLPTNITVLNLT  60
TLR3_Deer_Haplotype1    MSRPLPYHIYFFSGLLTCWILCTSSANKCTVRHEVADCSHLKLTQIPDDLPTNITVLNLT  60
                        * : *   *:*:.***   :**:**: **** *************:**************

TLR3_Human              HNQLRRLPAANFTRYSQLTSLDVGFNTISKLEPELCQKLPMLKVLNLQHNELSQLSDKTF  119
TLR3_Cattle             HNQLRRLPPANFTRYSQLTTLDGGFNSISKLEPELCQSLPWLEILNLQHNEISQLSDKTF  120
TLR3_Deer_Haplotype1    HNQLRRLPPANFTRYSQLTILDGGFNSISKLEPELCQSLPWLEVLNLQHNEISQLSDKTF  120
                        ******** ********** ** ***:**********.** *::*******:********

TLR3_Human              AFCTNLTELHLMSNSIQKIKNNPFVKQKNLITLDLSHNGLSSTKLGTQVQLENLQELLLS  179
TLR3_Cattle             IFCMNLTELHLMSNSIQKIKNDPFKNLKNLIKLDLSHNGLSSTKLGTQLQLENLQELLLS  180
TLR3_Deer_Haplotype1    IFCMNLTELHLMSNSIQKIQNDPFKNLKNLIKLDLSHNGLSSTKLGTQLQLENLQELLLS  180
                         ** ***************:*:** : ****.****************:***********

TLR3_Human              NNKIQALKSEELDIFANSSLKKLELSSNQIKEFSPGCFHAIGRLFGLFLNNVQLGPSLTE  239
TLR3_Cattle             NNKISSLTPGEFDFLGNSSLKRLELSSNQIKEFSPGCFHTLGELSGLSLNNAKLSPSLTE  240
TLR3_Deer_Haplotype1    NNKISSLTPEELDFLGNSSLKRLELSSNQIKEFSPGCFHAIGKLSGLSLNNAKLSPSLIE  240
                        ****.:*.  *:*::.*****:*****************::*.* ** ***.:*.*** *

TLR3_Human              KLCLELANTSIRNLSLSNSQLSTTSNTTFLGLKWTNLTMLDLSYNNLNVVGNDSFAWLPQ  299
TLR3_Cattle             KLCLELSNTSIENLSLSSNQLDTISHTTFDGLKQTNLTTLDLSRNSLRVMGNDSFAWLPH  300
TLR3_Deer_Haplotype1    KLCLELSNTSIENLSLSSNQLDTISHMTFDGLKQTNLTTLDLSRNSLRVMGNDSFAWLPH  300
                        ******:****.*****..**.* *: ** *** **** **** *.*.*:*********:

TLR3_Human              LEYFFLEYNNIQHLFSHSLHGLFNVRYLNLKRSFTKQSISLASLPKIDDFSFQWLKCLEH  359
TLR3_Cattle             LEYLSLEYNNIEHLSSRSFYGLSNLRRLDLRRSFTRQSISLTSLPKIDDFSFQWLKCLEY  360
TLR3_Deer_Haplotype1    LEYLFLEYNNIEHLSSRSFYGLSSLRHLNLRRSFTRQSISLTSLPKIDDFSFQWLKCLEY  360
                        ***: ******:** *:*::** .:* *:*:****:*****:*****************:

TLR3_Human              LNMEDNDIPGIKSNMFTGLINLKYLSLSNSFTSLRTLTNETFVSLAHSPLHILNLTKNKI  419
TLR3_Cattle             LNMDDNNFPGIKRNTFTGLVRLKFLSLSNSFSSLRTLTNETFLSLAGCPLLLLDLTKNKI  420
TLR3_Deer_Haplotype1    LNMEDNNFPGIKRNTFTGLVRLKFLSLSDSFSSLRTLTNETFLSLAGSPLLLLNLTKNKI  420
                        ***:**::**** * ****:.**:****:**:**********:*** .** :*:******

TLR3_Human              SKIESDAFSWLGHLEVLDLGLNEIGQELTGQEWRGLENIFEIYLSYNKYLQLTRNSFALV  479
TLR3_Cattle             SKIQSGAFSWLGHLEVLDLGLNEIGQELTGQEWRGLDNIVEIYLSYNKYLELTTNSFTSV  480
TLR3_Deer_Haplotype1    SKIQSGAFSWLGHLEMLDLGLNEIGQELTGQEWKGLDNIVELYLSYNKYLELTTNSFTSV  480
                        ***:*.*********:*****************:**:**.*:********:** ***: *

TLR3_Human              PSLQRLMLRRVALKNVDSSPSPFQPLRNLTILDLSNNNIANINDDMLEGLEKLEILDLQH  539
TLR3_Cattle             PSLQRLMLRRVALKNVDCSPSPFRPLPNLVILDLSNNNIANINDELLKGLEKLEILDLQH  540
TLR3_Deer_Haplotype1    PSLQRLMLRRVALKHVDSSPSPFHPLLNLVILDLSNNNIANINDELLKGLEKLEILDLQH  540
                        **************:**.*****:** **.**************::*:************

TLR3_Human              NNLARLWKHANPGGPIYFLKGLSHLHILNLESNGFDEIPVEVFKDLFELKIIDLGLNNLN  599
TLR3_Cattle             NNLARLWKHANPGGPVQFLKGLFHLHILNLGSNGFDEIPVEAFKDLRELKSIDLGMNNLN  600
TLR3_Deer_Haplotype1    NNLARLWKHANPGGPVQFLKGLSRLHILNLESNGFDEIPVEAFKDLRELKSIDLGMNNLN  600
                        ***************: ***** :****** **********.**** *** ****:****

TLR3_Human              TLPASVFNNQVSLKSLNLQKNLITSVEKKVFGPAFRNLTELDMRFNPFDCTCESIAWFVN  659
TLR3_Cattle             ILPQSVFDNQVSLKSLSLQKNLITSVQKTVFGPAFRNLSYLDMRFNPFDCTCESIAWFVN  660
TLR3_Deer_Haplotype1    ILPQSVFDNQVSLKSLRLQKNLITSVEKTVFGLAFRNLNYLDMSFNPFDCTCESIAWFVN  660
                         ** ***:******** *********:*.*** *****. *** ****************

TLR3_Human              WINETHTNIPELSSHYLCNTPPHYHGFPVRLFDTSSCKDSAPFELFFMINTSILLIFIFI  719
TLR3_Cattle             WINITHTNISELSNHYLCNTPPQYHGYPVMLFDVSPCKDSAPFELLFMININILLIFIFI  720
TLR3_Deer_Haplotype1    WINSTHSNISELRNHYLCNTPPQYHGFPVVLFDVSACKDSAPFELLFMISTNILLIFIFI  720
                        *** **:** ** .*******:***:** ***.* *********:***. .********

TLR3_Human              VLLIHFEGWRISFYWNVSVHRVLGFKEIDRQTEQFEYAAYIIHAYKDKDWVWEHFSSMEK  779
TLR3_Cattle             VLLIHFEGWRISFYWNVSVHRVLGFKEIDR-AEQFEYAAYIIHAYKDRDWVWKHSSPMED  779
TLR3_Deer_Haplotype1    VLLIHFEGWRISFYWNVSVHRVLGFKEIDR-AEQFEYAAYIIHAYKDRDWVWKHFSPMEE  779
                        ******************************  :***************:****:* * **.

TLR3_Human              EDQSLKFCLEERDFEAGVFELEAIVNSIKRSRKIIFVITHHLLKDPLCKRFKVHHAVQQA  839
TLR3_Cattle             EDHTLRFCLEERDFEAGVLELEAIVNSIRRSRKIIFVVTQNLLKDPLCKRFKVHHAVQQA  839
TLR3_Deer_Haplotype1    EDHTLRFCLEERDFEAGVFELEAIVNSIRRSRKIIFIITQNLLKDALCKRFKVHHAVQQA  839
                        **::*:************:*********:*******::*::**** **************

TLR3_Human              IEQNLDSIILVFLEEIPDYKLNHALCLRRGMFKSHCILNWPVQKERIGAFRHKLQVALGS  899
TLR3_Cattle             IEQNLDSIILIFLEEIPDYKLNHALCLRRGMFKSHCILNWPVQKERVNAFHHKLKVALGS  899
TLR3_Deer_Haplotype1    IEQNLDSIILIFLEEIPDYKLNHALCLRRGMFKSHCILNWPVQKERVNAFYHKLKVALGS  899
                        **********:***********************************:.** ***:*****

TLR3_Human              KNSVH 904
TLR3_Cattle             RNSAH 904
TLR3_Deer_Haplotype1    RNSVH 904
                        :**.*
```

Table S1. PCR cycling

Temperature	Exons 2 and 5	Exon 4	Cycle numbers
95˚C	9 min 45 sec	9 min 45 sec	
94˚C	20 sec	20 sec	3 cycles
60˚C	30 sec	30 sec	
72˚C	1.5 min	3 min	
94˚C	20 sec	20 sec	5 cycles
58˚C	30 sec	30 sec	
72˚C	1.5 min	3 min	
94˚C	20 sec	20 sec	5 cycles
56˚C	30 sec	30 sec	
72˚C	1.5 min	3 min	
94˚C	20 sec	20 sec	5 cycles
54˚C	30 sec	30 sec	
72˚C	1.5 min	3 min	
94˚C	20 sec	20 sec	5 cycles
52˚C	30 sec	30 sec	
72˚C	1.5 min	3 min	
94˚C	20 sec	20 sec	22 cycles
50˚C	30 sec	30 sec	
72˚C	1.5 min	3 min	
72˚C	7 min	7 min	
